# Refining the Genetic Contribution to Type 2 Diabetes Subtypes

**DOI:** 10.1111/dom.71159

**Published:** 2026-07-30

**Authors:** Nayra M. Al‐Thani, Salman M. Toor, Umm‐Kulthum Ismail Umlai, Usama Aliyu, Karsten Suhre, Omar M. E. Albagha

**Affiliations:** ^1^ College of Health and Life Sciences (CHLS) Hamad Bin Khalifa University (HBKU) Doha Qatar; ^2^ Bioinformatics Core Weill Cornell Medicine‐Qatar, Education City Doha Qatar; ^3^ Diabetes Research Centre (DRC), Qatar Biomedical Research Institute (QBRI) Hamad Bin Khalifa University (HBKU) Doha Qatar; ^4^ Englander Institute for Precision Medicine Weill Cornell Medicine New York New York USA

**Keywords:** GWAS, MOD, PGS, SIDD, SIRD, type 2 diabetes

## Abstract

**Background:**

Type 2 diabetes (T2D) is a complex and highly heterogeneous disease driven in part by genetic predisposition and can be stratified into clinical subgroups to aid disease management. We recently grouped T2D subjects in the Qatar Biobank (QBB) cohort into Severe Insulin‐Deficient Diabetes (SIDD), Severe Insulin‐Resistant Diabetes (SIRD), Mild Obesity‐Related Diabetes (MOD) and Mild Age‐Related Diabetes (MARD) subtypes. Herein, we focused on the genetic makeup of these subtypes.

**Methods:**

We used the QBB cohort (*n* = 13,808), of whom 2687 were with T2D, and comprehensively assessed polygenic risk scores (PGS) across T2D subtypes, investigated genetic loci associated with each subtype by leveraging the most recent and largest GWAS for T2D, evaluated SNP associations across T2D genetic clusters, and identified protein interaction pathways associated with these distinct T2D subtypes.

**Results:**

MOD showed consistently lower PGS compared with other T2D subtypes across all tested scores. SIDD showed more associations with SNPs mapping to residual glycemic cluster compared with other T2D subtypes. The incremental analysis of PGS004838 demonstrated a high ΔAUC of 0.101 for SIDD and a moderate ΔAUC of 0.068 for SIRD, but not for MOD and MARD. Protein interaction analyses identified candidate subtype‐associated gene networks linked to pathways related to glucose homeostasis in SIDD, insulin signalling and hepatic metabolism in SIRD, body fat distribution in MOD and vascular‐related processes in MARD.

**Conclusion:**

We found heterogeneous genetic architectures across clinically defined T2D subtypes in a Middle Eastern population. Our findings provide evidence supporting differential polygenic burden, subtype genetic associations and subtype‐associated biological pathways across T2D subtypes. These observations support the utility of subtype‐based genetic analyses for improving biological understanding of T2D heterogeneity.

## 1 Introduction

Type 2 diabetes (T2D) is a heterogeneous condition that involves an interplay of various genetic and environmental factors. Recent estimates have reported that diabetes affects ~1 in 9 adults globally, corresponding to more than 500 million individuals [1]. Efforts to tackle the global burden of diabetes primarily focus on lifestyle modifications. Individuals in the lifestyle intervention group showed a reduction in diabetes incidence of more than 50%, compared to ~30% in those receiving metformin in the Diabetes Prevention Program (DPP) study [2]. However, genetic predisposition remains a key determinant of diabetes risk and management, since it can influence both disease susceptibility and response to lifestyle interventions.

Large‐scale studies have uncovered the genetic risk of T2D, from candidate gene and positional cloning approaches to advancing through genome‐wide association studies (GWAS) [3, 4] that have identified hundreds of risk loci in key T2D‐associated genes, such as *TCF7L2*, *PPARG* and *FTO* [5]. However, over the 15 years since the pioneering GWAS on T2D, most associations have been reported in European populations and explain only a little proportion of the heritability. Alongside advances in genetic discovery, subclassifying T2D into more homogeneous subgroups better reflects disease heterogeneity [6].

Two subclassification methods for T2D were proposed in 2018, classifying T2D into four subtypes based on measurable variables [7] and associated genetic aberrations [8]. The first approach classified T2D into four subtypes: Severe Insulin‐Deficient Diabetes (SIDD), Severe Insulin‐Resistant Diabetes (SIRD), Mild Obesity‐related Diabetes (MOD) and Mild Age‐Related Diabetes (MARD) [7]. This classification successfully predicted disease progression and risk of complications, enabling a more tailored approach for patient management [9]. The second approach classified T2D into five clusters: Beta‐Cell, Proinsulin, Obesity, Lipodystrophy and Liver clusters by relying on soft clustering for 94 variants and 47 traits associated with T2D, which yielded clusters supported by mechanistic pathways [8]. This method was later expanded to include 300 variants (64 traits) and 650 variants (110 traits), resulting in 10 and 12 genetic clusters, respectively [10, 11]. Recently, Suzuki et al. devised 8 genetic clusters based on 1289 index SNPs associated with T2D across multiple ancestries [3]. The eight genetic clusters described by Suzuki et al. include the Beta‐cell +PI and Beta‐cell −PI clusters, both characterized by impaired β‐cell function, reduced fasting insulin secretion and increased glycemia. These clusters differ primarily in circulating proinsulin levels, with the Beta‐cell +PI cluster exhibiting elevated proinsulin concentrations and the Beta‐cell −PI cluster exhibiting reduced proinsulin concentrations, suggesting distinct mechanisms of β‐cell dysfunction. The Lipodystrophy cluster is characterized by insulin resistance, abnormal fat distribution and increased blood pressure, whereas the Liver/Lipid Metabolism cluster is associated with hepatic lipid metabolism and increased liver fat. The Obesity cluster is characterized by higher BMI and overall adiposity, while the Body Fat cluster is associated with variation in body fat composition and distribution. The two newly identified clusters include the Metabolic Syndrome cluster, characterized by higher fasting glucose, elevated triglycerides, lower HDL cholesterol and features of insulin resistance, and the Residual Glycemic cluster, which is associated predominantly with higher HbA1c and residual genetic effects on glycemic regulation not captured by the other clusters.

T2D subtypes proposed by Ahlqvist et al. [7], have shown differences in cardiovascular disease (CVD) risk. MOD was associated with lower CVD risk and higher estimated Glomerular filtration rate (eGFR) levels [12]. T2D subtype stratification in British Pakistani and Bangladeshi individuals showed improved T2D prediction when PGS were combined with a clinical risk prediction tool (QDiabetes) [13]. Additionally, T2D subclassifications have identified SIRD with the highest risk of Metabolic Dysfunction‐Associated Steatotic Liver Disease (MASLD) in European Belgian and Middle Eastern Qatari populations [14, 15]. In addition, SIDD and MOD previously showed higher genetic risk scores (GRS) compared to SIRD and MARD subtypes in European populations [16]. In contrast, SIDD had lower GRS compared to other T2D subtypes in the Indian population [17]. Moreover, a recent study of the Middle Eastern population showed that MARD had a distinct molecular profile linked to lipid metabolism, inflammation and aging, identified through the integration of multi‐omics platforms [18]. Another study in the European population has shown that T2D subtypes differ in body fat distribution between SIDD and SIRD [19]. Overall, several studies have reported genetic heterogeneity in T2D across populations [20, 21]. However, Middle Eastern populations are largely underrepresented.

GWAS conducted on T2D in the QBB cohort replicated several T2D‐associated loci and identified novel susceptibility loci in a multi‐ethnic meta‐analysis [22], defining the genetic architecture of T2D. Additionally, the loci showed allele frequencies concordant with those of European populations. However, the European‐derived PGS showed suboptimal performance in the Qatari population for many clinically relevant traits [23]. Moreover, using multi‐ancestry PGS rather than European‐derived PGS yielded higher predictive performance in the Qatari population [22]. We recently tested 140 T2D PGS, identifying the top‐performing scores for predicting prediabetes and progression to T2D in the QBB cohort [24].

In this study, we leveraged previously developed T2D PGS, evidence from the largest multi‐ancestry T2D GWAS, and our recent findings on T2D subtypes in the QBB cohort [14] to refine subtype‐enriched genetic contribution to T2D and highlight potential pathway differences in the pathophysiology of each subtype. Further investigations are needed to dissect the contribution of each association to the etiopathogenesis of each T2D subtype for clinical translation and improved disease management.

## 2 Methods

### 2.1 Study Population

The study cohort (*n* = 13,808) comprised participants from the Qatar Biobank (QBB), of whom 2687 were with T2D, enrolled as previously described [14]. Briefly, the QBB cohort comprises adult (≥ 18 years) Qatari citizens with comprehensive phenotypic, clinical and biological data, and biological samples. The study was conducted under ethical approval from the Institutional Review Board of Qatar Precision Health Institute (QPHI), Doha, Qatar (Protocol no. E‐2019‐QF‐QBB‐RES‐ACC‐0179‐0104) and Hamad Bin Khalifa University (QBRI‐IRB 2021‐03‐078). All participants provided written informed consent prior to participation in the study.

### 2.2 Data Curation

T2D subtypes were derived as described in the previous study [14], which replicated previously devised T2D stratification into subgroups [7]. Whole‐genome sequencing (WGS) was conducted as part of the Qatar Genome Project (QGP) for 14,409 individuals from the QBB [25]. Additional downstream quality‐control measures using PLINK and Hail [23] led to the retention of 13,808 high‐quality samples for association analysis.

### 2.3 Assessment of T2D Subtypes by Using PolyGenic Risk Score (PGS)

All PGS used herein were downloaded from the PGS Catalogue (https://www.pgscatalog.org) and have been previously tested for predicting prediabetes and progression to T2D in the QBB cohort [24]. The 140 PGS were scaled to a mean of 0 and a standard deviation of 1. We assessed the performance of the 140 PGS for predicting each T2D subtype from healthy controls (*n* = 8671) using logistic regression analyses adjusted for age, sex and the first 10 genetic principal components. The top five best‐performing PGS were selected (Table S1) for each subtype; because several PGS overlapped across subtypes, this resulted in nine unique PGS used in downstream analyses. These include: PGS004838 [26] in SIDD, SIRD, MOD and MARD; PGS004859 [27] in SIDD, SIRD and MOD; PGS004837 [26] in SIDD, SIRD and MARD; PGS002308 [28] in SIDD, MOD and MARD; PGS004887 [29] in SIDD, SIRD and MOD; PGS003443 [30] in SIRD; PGS004870 [31] in MOD; whereas the last two PGS003867 [32] and PGS002771 [33] in MARD. The top PGS for T2D subtypes were then evaluated for their ability to discriminate between T2D subtypes using logistic regression and the area under the curve (AUC) in the Receiver Operating Characteristic (ROC) curve analysis of the test dataset.

### 2.4 Statistical Testing and Model Prediction

PGS were evaluated across T2D subtypes. Statistically significant differences among T2D subtypes in the top 5 PGS for each subtype were assessed by using a pairwise Wilcoxon rank sum test with Benjamini‐Hochberg adjustment.

For each PGS, individuals were divided into quartiles to assess the proportion of T2D subtypes across PGS risk groups, ranging from lower risk (quartile 1; Q1) to higher risk (quartile 4; Q4). The Cochran‐Armitage trend test was used to assess linear trends in subtype‐specific proportions across PGS quartiles. Results are presented as *Z*‐scores with *p‐*values for each T2D subtype. A positive *Z*‐score indicates a higher proportion in the highest PGS quartile, while a negative *Z*‐score indicates a lower proportion in the highest PGS quartile.

Generalized linear models were used to assess the performance of each PGS in predicting T2D subtype (one subtype vs. others), with the data split into 60% training and 40% testing subsets. Analyses for genetic risk stratification and assessment of predictive performance were conducted in R (v4.3.1). The following models were evaluated:

$$\text{Model}-1=\left(T2D-\text{subtypes}\sim \mathrm{Age}\right)$$

$$\text{Model}-2=\left(T2D-\text{subtypes}\sim \text{Gender}\right)$$

$$\text{Model}-3=\left(T2D-\text{subtypes}\sim \mathrm{BMI}\right)$$

$$\text{Model}-4=\left(T2D-\text{subtypes}\sim \mathrm{Age}+\mathrm{BMI}\right)$$

$$\text{Model}-5=\left(T2D-\text{subtypes}\sim \mathrm{PGS}\right)$$

$$\text{Model}-6=\left(T2D-\text{subtypes}\sim \mathrm{Age}+\mathrm{PGS}\right)$$

$$\text{Model}-7=\left(T2D-\text{subtypes}\sim \mathrm{BMI}+\mathrm{PGS}\right)$$

$$\text{Model}-8\;\left(T2D-\text{subtypes}\sim \mathrm{Age}+\mathrm{BMI}+\mathrm{PGS}\right)$$

ROC curve analysis was performed and the AUC was calculated for all models. The incremental improvement in discrimination (ΔAUC) attributable to PGS was quantified as the difference in AUC between the Age + BMI model (Model 4) and the Age + BMI + PGS model (Model 8). This comparison was used to assess the additional predictive value of incorporating the PGS for discriminating between T2D subtypes. To assess whether the observed improvement in discrimination with the addition of the PGS was statistically significant, paired DeLong tests were conducted to compare the ROC curves of Models 4 and 8. The DeLong test determines whether the observed difference in AUC exceeds what is expected by sampling variability.

### 2.5 Assessment of T2D Subtypes Through Targeted Association Analysis of Known T2D Loci

Assessment of the genetic contribution to T2D subtypes involved performing targeted association analyses across T2D subtypes, based on previously known T2D‐associated SNPs from the largest and most recent GWAS previously conducted on T2D [3, 4]. We conducted targeted association analyses using the SNPs previously known to be associated with T2D in studies by Mahajan et al. [4], and Suzuki et al. [3]. The scalable and accurate implementation of the generalized mixed model (SAIGE) was used with adjustment for covariates, including age, sex, BMI and principal components (PC1–PC4). The analysis relied on the BMI‐adjusted model, as the targeted association analyses were restricted to T2D individuals only. Furthermore, to identify the potential genetic association of T2D SNPs across subtypes, 10 targeted association analyses were conducted: 1‐ SIDD (cases) versus other T2D subtypes as controls: including SIRD, MOD, MARD; 2‐ SIRD (cases) versus other T2D subtypes as controls; 3‐ MOD (cases) versus other T2D subtypes as controls; 4‐ MARD (cases) versus other T2D subtypes as controls. Analyses 5–10 included subtype based paired comparisons: 5‐ SIDD (cases) versus SIRD (controls); 6‐ SIDD (cases) versus MOD (controls); 7‐ SIDD (cases) versus MARD (controls); 8‐ SIRD (cases) versus MOD (controls); 9‐ SIRD (cases) versus MARD (controls); 10‐ MOD (cases) versus MARD (controls). A coverage heatmap was generated for all captured SNPs and significant SNPs across the 10 analyses.

### 2.6 Refining Genetic Clusters and Pathway Analysis of T2D Subtypes

Statistically significant SNPs obtained from targeted association analyses were curated and mapped to corresponding genes. SNP annotations were performed using SNPnexus (https://www.snp‐nexus.org/v4/). Variants from targeted association analyses were used to map subtype‐associated variants to the 8 functional genetic clusters previously reported by Suzuki et al. [3]. The clusters include Beta‐cell −PI, Beta‐cell +PI, Body fat, Lipodystrophy, Liver/lipid metabolism, Metabolic syndrome, Obesity and Residual glycemic.

Protein interaction pathway analyses were performed using Ingenuity Pathway Analysis (IPA) software (Qiagen) to identify potential biological networks and pathways associated with the different T2D subtypes. The ‘Core Analysis’ function using default parameters was performed to identify canonical pathways and protein interaction networks associated with Diabetes mellitus. A broader protein–protein interaction (PPI) network from the OmniPath database [34] was also generated using the OmnipathR package in R (v4.3.1). We focused on direct interaction networks to include all known interactions with the interacting partner from T2D subtypes gene list, followed by focusing on the protein network for the subtype‐enriched (SIDD, SIRD, MOD and MARD) interactions. The PPI pairs were exported for visualization using Cytoscape software [35].

## 3 Results

### 3.1 Polygenic Risk Score Assessment of T2D Subtypes in the QBB Cohort

**FIGURE 1:**
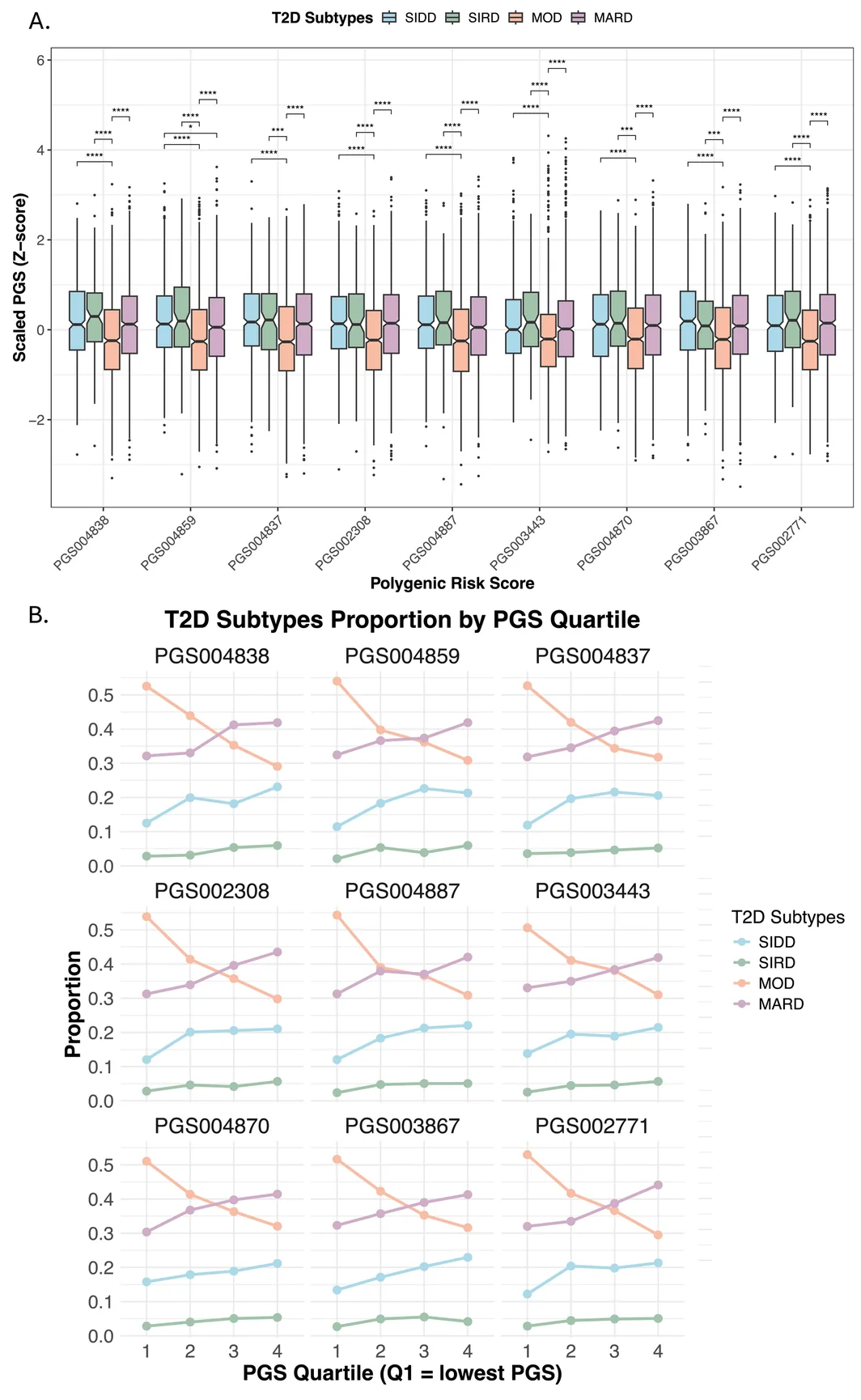
(A) Polygenic risk score (PGS) evaluation of T2D subtypes. The box plot shows the distribution of PGS scores across QBB T2D subtypes (SIDD, SIRD, MOD, MARD) using the top 5 best‐performing PGS for each subtype (Table S1). Because several PGS overlapped across subtypes, this resulted in nine unique PGS used in the analyses and are shown here. The boxes represent the interquartile range and the *y*‐axis represents the scaled PGS (mean = 0, standard deviation = 1). FDR‐adjusted *p‐*value from Wilcoxon rank‐sum test: * < 0.05; ** < 0.01; *** < 0.001; **** < 0.0001. (B) Risk stratification of T2D subtypes based on PGS quartiles. Line graphs show the proportion of T2D subtypes across PGS genetic risk quartiles using the best‐performing PGS. The Cochran‐Armitage test was used to assess linear trends in subtype‐specific proportion across PGS quartiles. Results were significant (*p*‐value < 0.05; Table S3) for all subtypes across the tested PGS except for SIRD in PGS003867 and PGS004837. The number of subjects in each subtype was SIDD (*n* = 495), SIRD (*n* = 116), MOD (*n* = 1,080), and MARD (*n* = 996).

The 140 PGS were assessed for their ability to predict T2D subtypes in the QBB cohort, in which we identified the best‐performing PGS for each subtype based on AUC (Table S1). The top performing 5 PGS for each T2D subtype (Table S1): PGS004838 in SIDD, SIRD, MOD and MARD; PGS004859 in SIDD, SIRD and MOD; PGS004837 in SIDD, SIRD and MARD; PGS002308 in SIDD, MOD and MARD; PGS004887 in SIDD, SIRD and MOD; PGS003443 in SIRD; PGS004870 in MOD; PGS003867 in MARD; PGS002771 in MARD were evaluated (Figure 1A). SIDD, SIRD and MARD presented with significantly higher PGS scores compared to MOD across all tested PGS, while no significant differences in PGS scores were recorded between SIDD, SIRD and MARD, except PGS004859, which showed marginal significance between SIDD and MARD (Figure 1A, Table S2). Next, we stratified T2D subtypes into quartiles based on PGS scores, with Q1 representing the lowest and Q4 the highest genetic risk (Figure 1B). SIDD, SIRD and MARD showed increasing subtype proportion across higher PGS quartiles, whereas MOD showed the highest T2D proportion in Q1 and the lowest in Q4 (Figure 1B, Table S3). A strong positive trend was prominent across all PGS for SIDD (PGS004859, *Z* = 5.06, *p‐*value = 4.08 × 10^−7^) and MARD (PGS002308, *Z* = 5.09, *p‐*value = 3.51 × 10^−7^) indicating a higher proportion among individuals in the top PGS quartiles. Moreover, a positive trend of increasing subtype proportion was observed in SIRD with all PGS (highest; PGS004838, *Z* = 3.32, *p*‐value = 9.10 × 10^−4^) except for PGS003867 and PGS004837 (Figure 1B, Table S3). In contrast, a strong negative trend was observed across all PGS for MOD, indicating higher proportion in the lowest PGS quartiles (highest; PGS004838, *Z* = −9.34, *p*‐value = 9.49 × 10^−21^).

**FIGURE 2:**
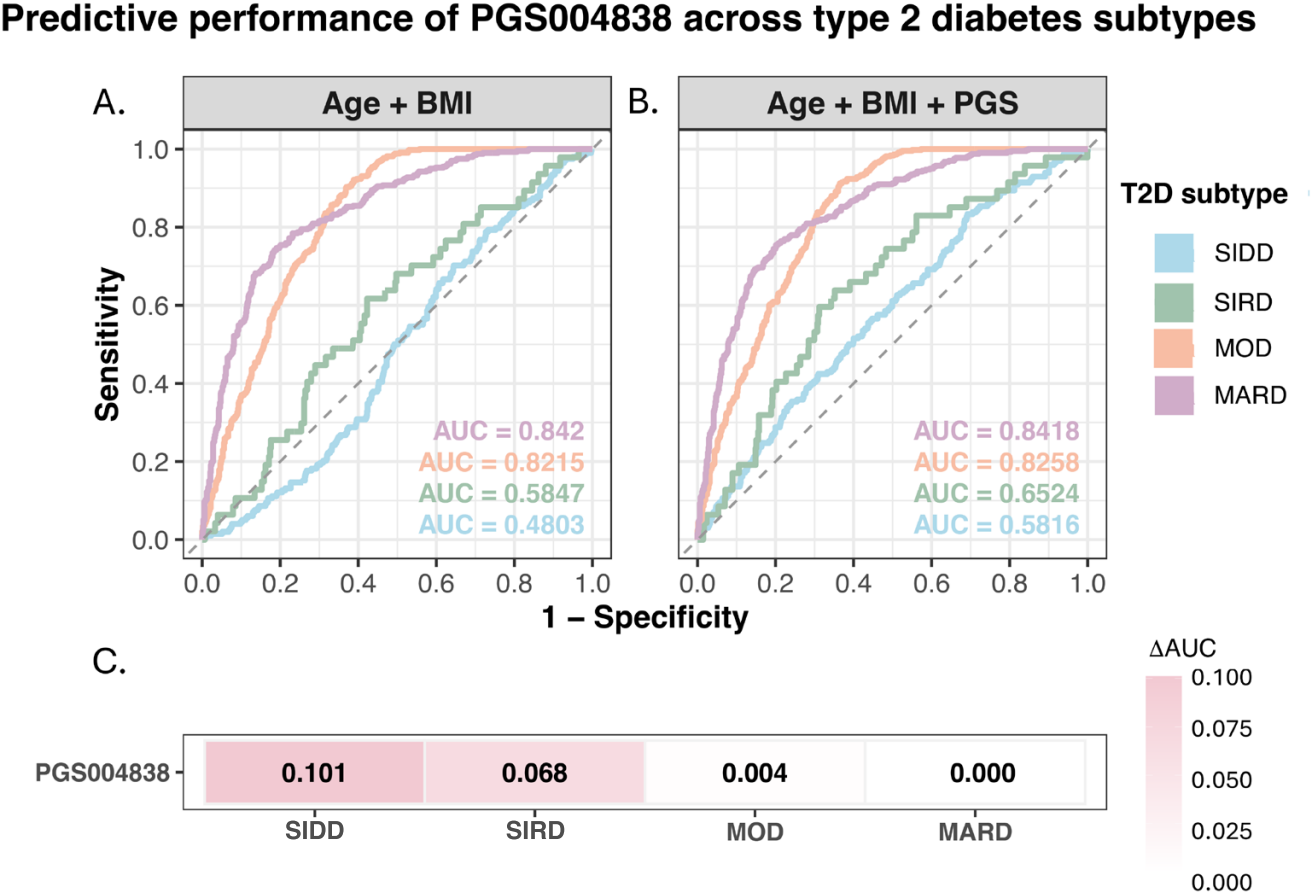
Predictive performance assessment of best‐performing PGS for T2D subtypes. Receiver Operating Characteristic (ROC) analysis illustrates the performance of PGS004838 in predicting T2D subtypes (SIDD, SIRD, MOD, MARD) using logistic regression in (A) Model 4 (age, BMI) and (B) Model 8 (age, BMI, PGS). The area under the curve (AUC) values are reported for each subtype. Sensitivity is shown on the *y*‐axis and 1‐Specificity on the *x*‐axis. (C) The heatmap shows the incremental improvement in model prediction when PGS is added and shown as Δ AUC of the comparisons between Models 4 and 8 for PGS004838.

Given significant differences in PGS scores across T2D subtypes, we then assessed the predictive performance of the PGS to discriminate between the subtypes (Table S4). Overall, ROC analysis across all models indicates that Age (Model‐1) exhibited a high AUC for MOD and MARD, but not for SIDD and SIRD (Figure S1). Conversely, Gender did not show much improvement in AUC; therefore, we did not test it with PGS, while BMI (Model‐3) demonstrated a modest AUC for SIRD, MOD and MARD, but not for SIDD. We then compared Model 4 (Age + BMI) with Model 8 (Age + BMI + PGS) to evaluate improvements in AUC when PGS were added to the models (Figure S1). The PGS004838, which was among the top five PGS shared across the four T2D subtypes, demonstrated a significant increase in AUC for SIDD (ΔAUC = 0.101) and SIRD (ΔAUC = 0.068) but not for MOD and MARD (Figure 2).

We then performed the DeLong test to assess the significance of incremental improvement for all tested PGS when added to Model 4. We found that the highest Δ AUC was for SIDD across the majority of the top PGS, including PGS004838 (Figure 2C), PGS004859, PGS004837, PGS002308, PGS004887 and PGS003867 (*p*‐value < 0.001; Table S4). While SIRD demonstrated modest significance in Δ AUC for PGS004838 (Figure 2C) and PGS003443 (Table S4). Interestingly, MOD exhibited a low or non‐significant Δ AUC, whereas MARD displayed non‐significant Δ AUC across all tested PGSs (Figure 2C; Table S4).

### 3.2 Assessment of T2D‐Associated SNPs in T2D Subtypes

We performed targeted association analyses across T2D subtypes using genome‐wide significant T2D‐associated SNPs from two large multi‐ancestry studies (Mahajan et al. [4] and Suzuki et al. [3]). The significantly associated SNPs (*n* = 249; *p‐*value < 0.05; Table S5) were used for downstream pathway analysis for T2D subtypes.

**FIGURE 3:**
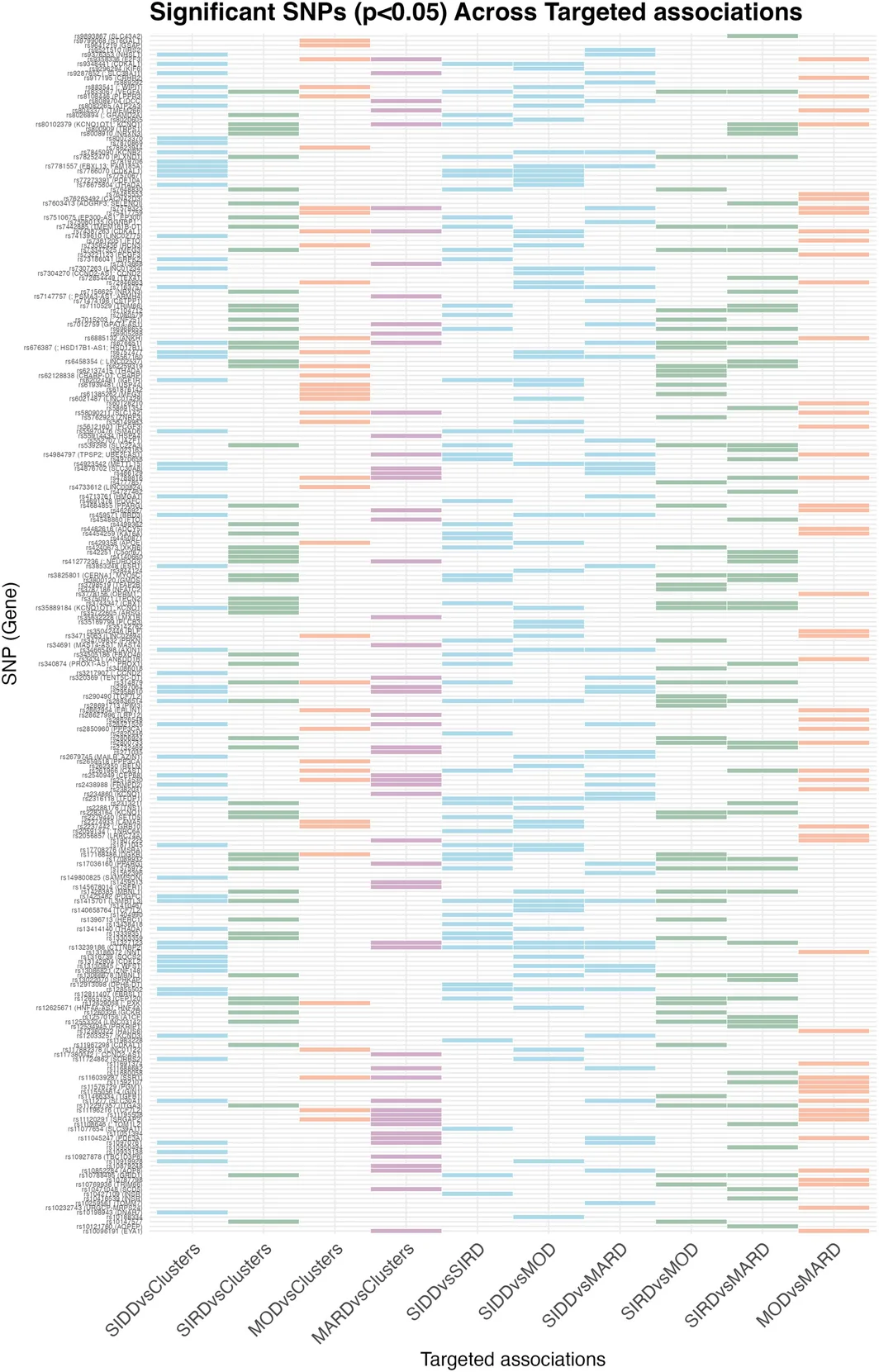
Associations of T2D‐associated genome‐wide significant SNPs with T2D subtypes. The matrix shows the results of targeted association analyses conducted on SNPS reported from Mahajan et al. [4], and Suzuki et al. [3]. SNPs that showed nominal statistical significance (*p‐*value < 0.05) with T2D subtypes in each targeted association analysis are shown. The *y*‐axis lists SNPs (Gene) and the *x*‐axis represents the different comparisons of the targeted association analyses.

Results of targeted association analyses are presented in Figure 3. Significant SNP associations were enriched in specific‐subtype targeted association analyses but not across all subtype comparisons (Figure 3). Some genes showed significant associations in SIDD but not in the other subtypes, such as *FBXL13, IGF1R, SMAD6, AXIN1* and *KCNB2* (Figure 3; Table S5). In SIRD, significant associations were observed for *SELENOI, NRXN3, HERC1* and *GCKR*. In MOD, significant associations were observed for *ERLIN1, HAUS6* and *PPP3CA*, whereas in MARD, significant associations were observed for *QSER1, MAST4* and *PDE3A*. Some associations were shared across subtypes: *KCNQ1* and *CDKAL1* were associated with all four T2D subtypes, whereas *PPARG, DGKB, PROX1, FTO, VEGFA* and *TCF7L2* were associated with three subtypes. We also performed pathway analysis to investigate associations among specific protein networks in each T2D subtype.

**FIGURE 4:**
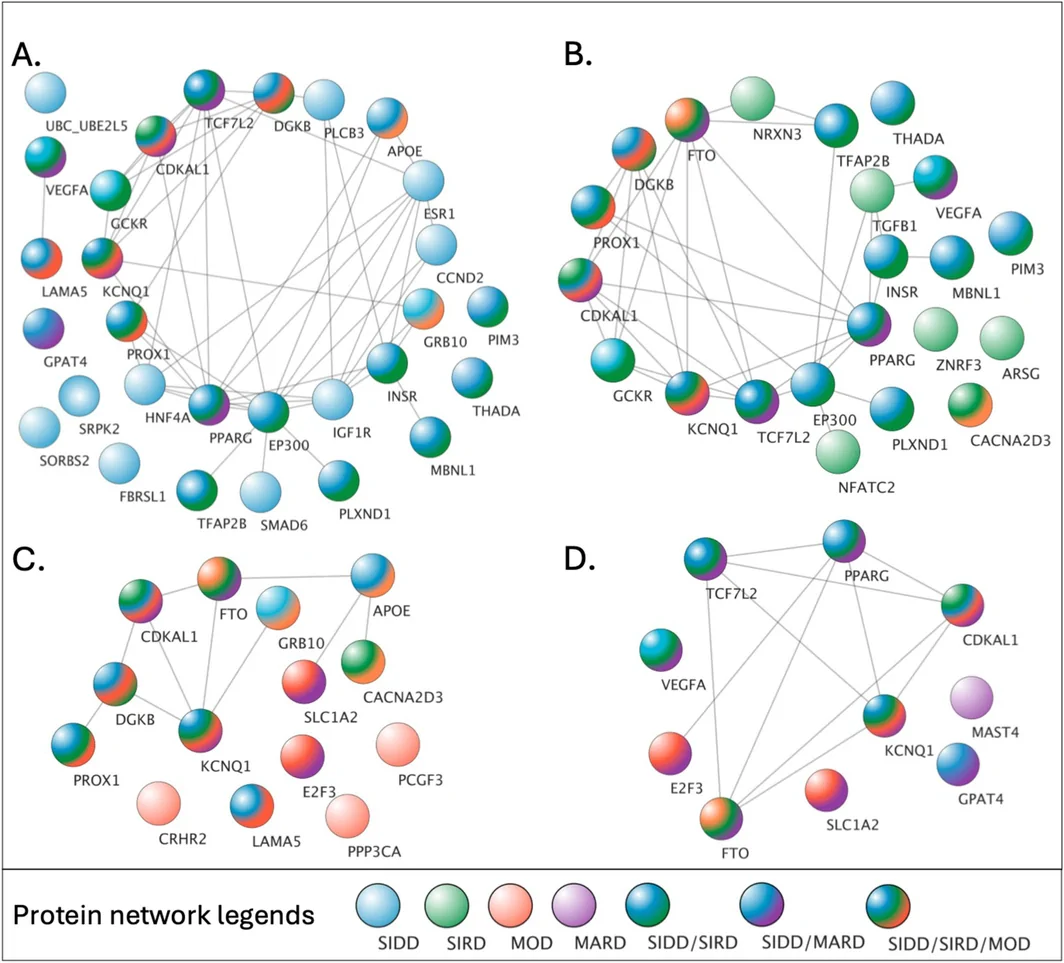
Protein‐interaction networks of T2D subtype‐associated proteins. The network shows analysis of candidate genes derived from annotated SNPs associated with T2D subtypes. (A) SIDD‐associated protein network linked to insulin signalling; (B) SIRD‐associated protein network correlated with insulin secretion and liver homeostasis; (C) MOD‐associated protein network affecting glucose metabolism and insulin sensitivity; (D) MARD‐associated proteins network correlated with cardiovascular complications. Encoded proteins (nodes) are colour‐coded to indicate their association with the corresponding T2D subtypes (SIDD: Blue; SIRD: Green; MOD: Orange; MARD: Purple). Overlapping protein associations in multiple T2D subtypes are overlaid with the colour of each T2D subtype.

We generated protein interaction networks from genes, enriched for variants in each T2D subtype identified from targeted association analyses (Figure 3). Results showed several T2D‐associated proteins with known biological functions associated with beta‐cell function, insulin signalling (sensitivity, secretion), energy metabolism (glucose, adipogenesis) and cardiovascular function (cardiac fibrosis, endothelial vascularization) (Figure 4; Figure S2). These proteins predominantly exhibited roles in affecting insulin signalling via *ARSG* and *NFATC2*, and were associated with Nonalcoholic fatty liver disease (NAFLD) through ZNRF3, *INSR, PPARG, FTO, GCKR* and *TFGB1* in SIRD. Beta‐cell function (*UBC, UBE2L5*) and insulin signalling pathways involving *PLCB3, CCND2* and *SRPK2* were observed in SIDD. In contrast, MOD showed association with lipid metabolism pathways, particularly involved in body fat distribution via *CDKAL1, KCNQ1, GRB10, APOE, DGKB, PCGF3* and *E2F3*. MARD showed association with vascular complications through the involvement of *TCF7L2, PPARG, FTO, KCNQ1, MAST4, VEGFA* and *CDKAL1* (Figure 4).

### 3.3 Genetic Clustering of T2D Subtypes

**FIGURE 5:**
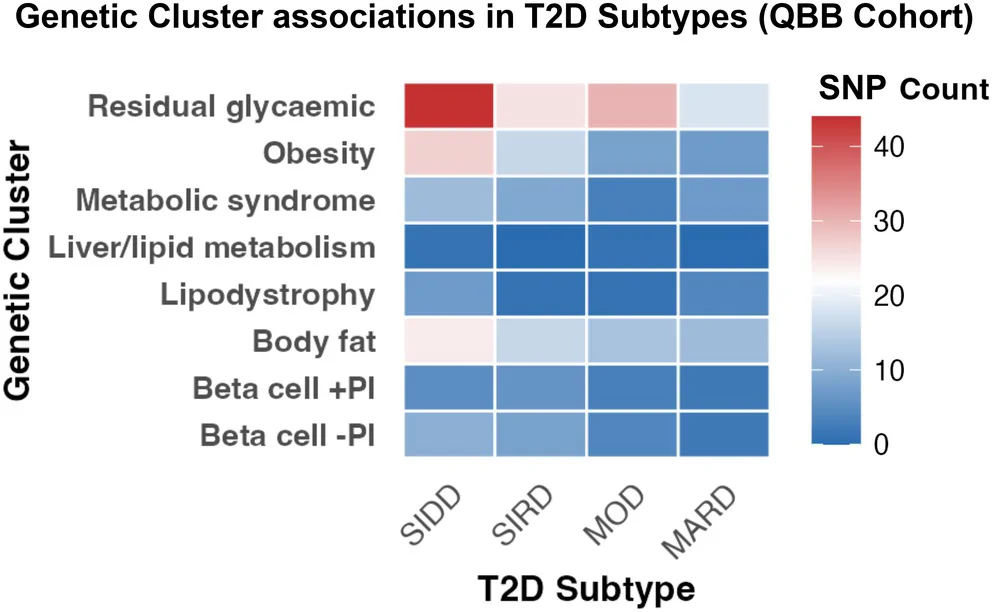
Genetic cluster associations in T2D subtypes. The heatmap shows SNP counts for variants associated with T2D subtypes corresponding to the 8 genetic clusters previously reported by Suzuki et al. [3]

For subtype‐specific genetic association, we followed the 8 genetic clusters reported previously by Suzuki et al. [3] All T2D subtypes were associated with the residual glycemic cluster, with SIDD showing a higher SNP count compared to other subtypes (Figure 5, Table S6). Additionally, SIDD showed a higher SNP count association with obesity and body fat clusters compared to the other T2D subtypes, whereas SIRD showed a modest SNP count association for obesity and body fat clusters (Figure 5). Overall, MOD and MARD showed fewer subtype‐associated SNPs across the genetic clusters than SIDD and SIRD.

## 4 Discussion

Previous studies have demonstrated T2D heterogeneity by classifying T2D into subtypes [7, 8], and conducted large multi‐ancestry GWAS to determine the underlying T2D‐associated genetic risk [3, 4]. The molecular pathways among T2D subtypes based on genetic aberrations have also been explored [11]. We focused on bridging these findings to explore the potential genetic contribution to the etiopathogenesis of T2D subtypes to improve understanding of T2D heterogeneity in an underrepresented population with high T2D prevalence. We evaluated the contribution of PGS across T2D subtypes, deciphered their underlying genetic profiles, and uncovered potential biological pathways across the different subtypes. To our knowledge, this is among the first comprehensive analyses integrating polygenic risk, targeted association analyses and genetic clustering to investigate T2D subtypes in a Middle Eastern population.

SIDD, SIRD and MARD showed significantly higher PGS compared with MOD individuals, consistent with previous studies reporting subtype‐specific differences in genetic risk burden [21]. Recent observations of MOD individuals showed a decline in BMI 10 years after diagnosis, indicating subtype‐specific metabolic trajectories [36]. In our study, we observed lower PGS in MOD, which suggests that genetic susceptibility may play a comparatively smaller role in the MOD subtype. Of note, these findings could be influenced by PGS derived from BMI‐adjusted GWAS, although information on BMI adjustment in source studies was limited and not conclusively available in the database. To the best of current knowledge, this is the first report indicating that MOD has a lower T2D‐related PGS compared to other subtypes.

The PGS used in the present study were derived from broad populations and have previously shown high predictive performance for prediabetes in the Qatari population [24]. The predictive performance of PGS improved with the inclusion of age and BMI. Overall, the predictive models performed best for MOD and MARD, which make up the majority of T2D cases within QBB [14]. However, this was driven mainly by age and BMI since the addition of PGS showed little discriminatory improvements. The Δ AUC analysis for including PGS in the model showed significant improvements in SIDD, and SIRD, but not in MOD and MARD subtypes. This suggests that genetic factors may play a larger role in SIDD and SIRD.

Using a targeted association analyses approach restricted to previously reported T2D‐associated loci, we identified subtype‐specific patterns of association involving well‐established T2D genes, including *TCF7L2, CDKAL1, KCNQ1, PPARG, FTO* and *GCKR*. While these associations were nominally significant and limited to known loci, their subtype‐specific distribution is consistent with prior large‐scale studies exploring genetic heterogeneity in T2D. Subsequent genetic cluster and protein interaction analyses highlighted pathways plausibly linked to beta‐cell dysfunction, insulin resistance, lipid metabolism and vascular biology.

Recent reports showed that T2D subtypes are genetically associated with multiple biological pathways that contribute to T2D heterogeneity, but not strongly with subtype‐specific pathways [37]. Previously, genotyping of 10 well‐established T2D‐associated genes in T2D showed stronger genetic associations with *TCF7L2, KCNQ1* and *CDKAL1* in the T2D subtypes SIDD and MOD [38]. In agreement with Ahlqvist and Mansour Aly et al., *TCF7L2* SNPs showed an association with SIDD, MOD and MARD, but not with SIRD in our cohort [7, 16]. *FTO* variants have been previously reported in the MOD cluster [7]. We found that *FTO* variants were associated with MOD, SIRD and MARD. *KCNQ1* is known to have a role in insulin secretion and an increased risk of nephropathy [39]. *KCNQ1* was associated with SIDD, SIRD and MARD, but with stronger significance in SIRD and MARD, suggesting *KCNQ1* could affect insulin secretion in these subtypes.

Gurung et al. reported 295 proteins that are related to neurogenesis, cell adhesion and inflammation, and showed distinct expression across T2D subtypes [40]. The protein network analysis indicated that SIDD‐specific pathogenesis is associated with insulin signalling, which includes *PLCB3* [41], *CCND2* [42], *IGF1R* [43] and *SRPK2* [44]. Moreover, its correlation with beta‐cell dysfunction involves ubiquitin activation component (UBC) [45], *UBC_UBE2L5*. SIRD‐specific network correlated with proteins related to liver homeostasis, such as *ZNRF3* [46], insulin secretion dysregulation through *ARSG* [47], and insulin resistance in *TGFB1* [48]. MOD has an association with *CRHR2* involved in glucose metabolism and insulin sensitivity [49], while MARD is associated with cardiovascular risk involving *VEGFA* [50]. Combined, our findings suggest distinct genetic underpinnings for each T2D subtype and support the utility of molecular clustering to guide stratified T2D interventions in the future.

Previous subclassification of T2D based on genetic association led to eight clusters [3]. Our analysis of these clusters in relation to the T2D subtypes showed more associations of the residual glycemic cluster in SIDD, followed by obesity and body fat clusters. Our findings reflect the driver interaction protein networks with the genetic associations for T2D subtypes. SIDD mapped to the residual glycemic cluster, supported by network pathways involved in glucose homeostasis. SIRD was linked to the body fat and obesity clusters, reflecting associations with obesity, insulin resistance and kidney complications, as reflected in protein interactions in pathways associated with insulin secretion and NAFLD. MOD and MARD showed a milder association with the genetic clusters. However, based on the pathway analysis, obesity‐related pathways in body fat distribution were observed in MOD, while pathways potentially related to age‐associated vascular consequences were observed in MARD.

One of the key limitations of the present study was the reliance on investigating genetic associations for T2D subtypes on previously known loci [3, 4], due to the limited sample size of the QBB cohort. In our targeted association analyses, the genes nearest to the T2D‐associated SNPs were annotated as putative effector genes. Although this is a common approach, many SNPs exert their effects by regulating distant genes, and the causal effector gene may differ from the nearest annotated gene. Moreover, we used previously known associations for the genetic clusters by relying on the strongest reported associations [8, 10, 11], which were only partially replicated in our targeted association analyses. Additionally, functional validation of components of potential networks associated with different T2D subtypes will aid in identifying robust variants for each subtype. Our targeted association findings are based on nominal significance and warrant validation in larger independent cohorts. Further, replication in an independent Middle Eastern or multi‐ancestry cohort would strengthen the genetic associations of T2D subtypes.

In summary, our study demonstrates that clinically defined T2D subtypes in the QBB cohort exhibit distinct patterns of polygenic burden, genetic association based on targeted analysis, within the genetic clusters and associated biological networks. By extending previous subtype‐genetic analyses to an underrepresented Middle Eastern population, these findings contribute to a more globally representative understanding of T2D genetic heterogeneity. While further functional studies are required, subtype‐informed genetic analyses provide a valuable framework for refining biological stratification of T2D.

## Author Contributions

N.M.A. data curation, investigation, formal analysis and writing – original draft. S.M.T. investigation and writing – review and editing. U.‐K.I.U/U.A. data curation, investigation. K.S. supervision, investigation and writing – review and editing. O.M.E.A. conceptualization, funding acquisition, formal analysis, supervision, investigation and writing – review and editing.

## Funding

This study was supported by a grant from the Qatar National Research Fund (QNRF) awarded to OMEA and KS (NPRP11C‐0115‐180010). Open access funding provided by the Qatar National Library.

## Conflicts of Interest

The authors declare no conflicts of interest.

## Supporting information


**Figure S1:** AUC Models for T2D subtypes using the top 5 PGS for each T2D subtype. Variables included in each model are shown on the right. The *x*‐axis and *y*‐axis represent 1—Specificity, and Sensitivity respectively.
**Figure S2:** Protein‐interaction Networks of T2D subtype‐associated proteins. The network shows analysis of candidate genes obtained from annotated genes from variants associated with each T2D subtype. Encoded proteins (nodes) are colour‐coded to indicate their associaFon with the corresponding T2D subtypes (SIDD: blue; SIRD: green; MOD: orange; MARD: purple). Overlapping protein associaFons in mulFple T2D subtypes are overlaid with the colour of each T2D subtype.

**Table S1:** The Top 5 Best‐Performing T2D Polygenic Risk Score in the QBB cohort assessed for discriminating diabetes subtypes from healthy controls.
**Table S2:** Comparisons of 5 best performing PGS across T2D Subtypes*.
**Table S3:** Cochran‐Armitage trend test of the proportion of T2D subtypes across PGS quartiles*.
**Table S4:** Incremental improvement (DeltaAUC) in T2D subtype prediction when PGS were added to Model 4 (Age + BMI).
**Table S5:** Targeted Association Analysis of preveiously reported T2D‐associated SNPs in the QBB cohort*.
**Table S6:** Index SNPs from Suzuki et al. [3] achieving nominal significant association in T2D subtypes presented across the 8 genetic clusters.

## Data Availability

The data analysed in this study can be obtained through an ISO‐certified protocol. Requests to access these datasets are subject to approval by the Institutional Review Board of the Qatar Precision Health Institute (QPHI), Qatar Foundation, Doha, Qatar, and should be directed to https://www.qphi.org.qa/research/how‐to‐apply.
