# Supplementary material for: Refining the Genetic Contribution to Type 2 Diabetes Subtypes
Source: Diabetes Obes Metab. 2026 Jul 30;28(10):9450–60. doi: 10.1111/dom.71159 (PMC13538756; doi:10.1111/dom.71159)
Supplement: Supplementary file 1 [file DOM-28-9450-s002.pdf]

Supplementary Figures:

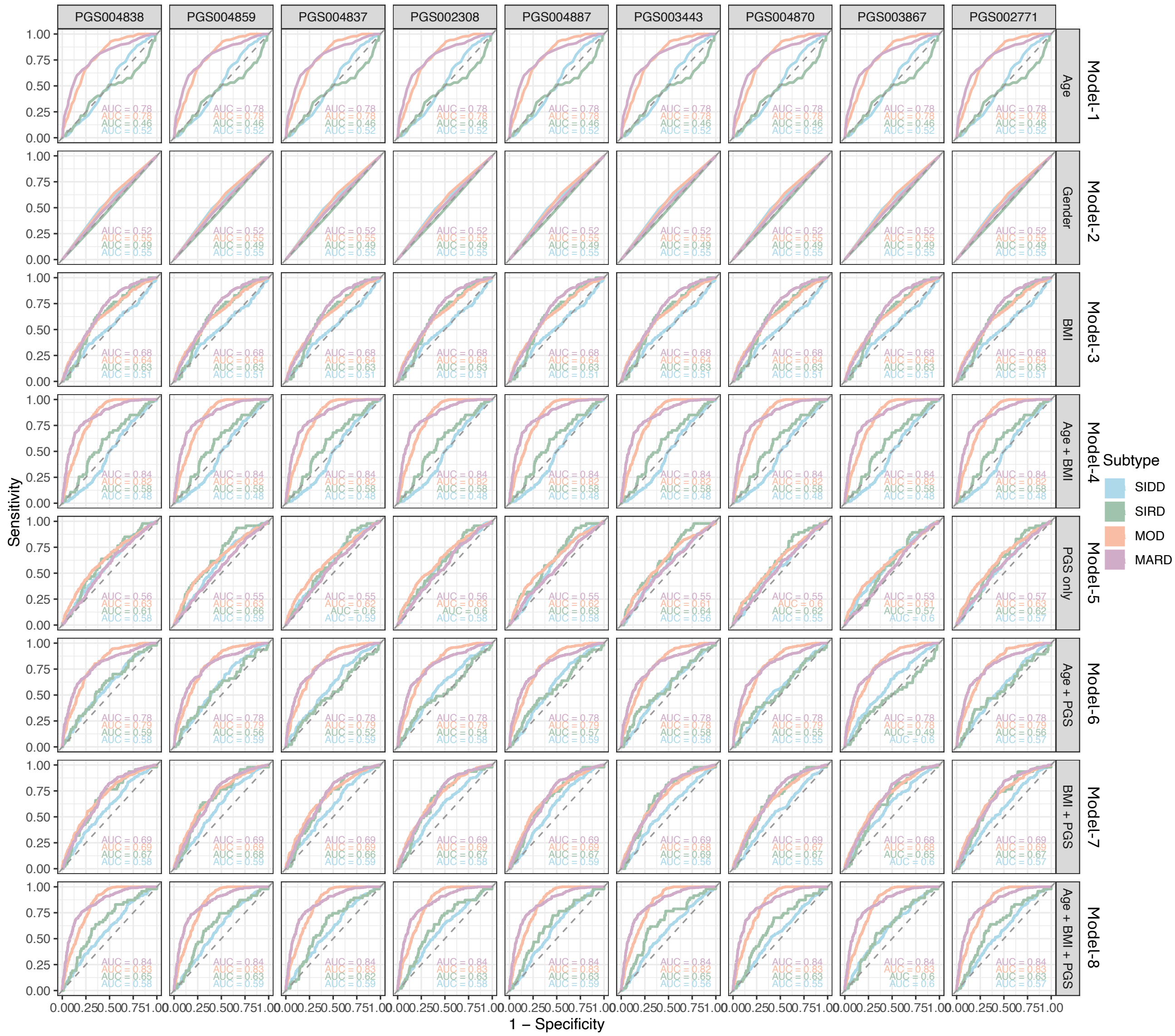

Figure S1: AUC Models for T2D subtypes using the top 5 PGS for each T2D subtype. Variables included in each model are shown on the right. The x-axis and y-axis represent 1 – Specificity, and Sensitivity respectively.

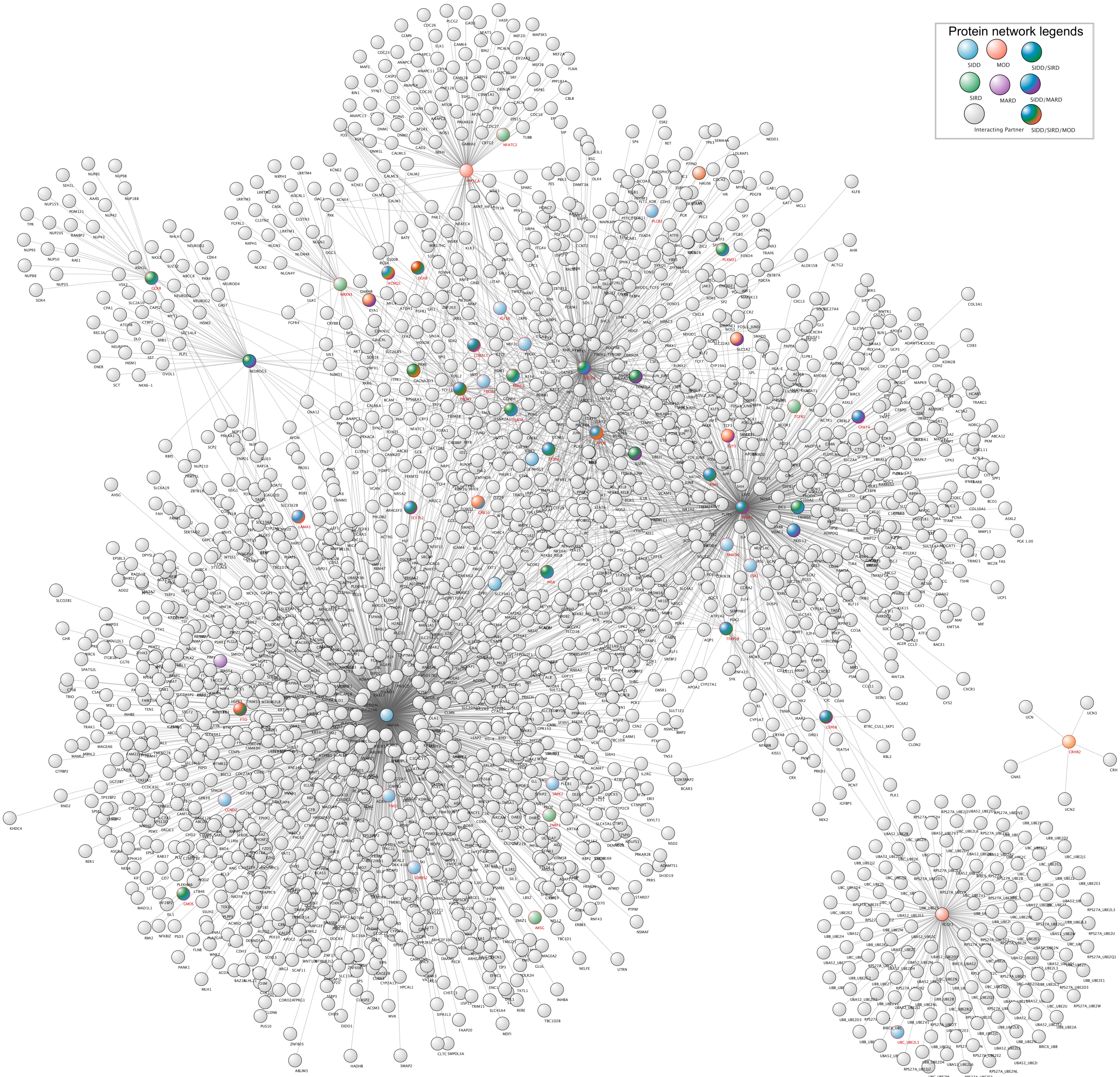

Figure S2: Protein-interaction Networks of T2D subtype-associated proteins. The network shows analysis of candidate genes obtained from annotated genes from variants associated with each T2D subtype. Encoded proteins (nodes) are color-coded to indicate their association with the corresponding T2D subtypes (SIDD: blue; SIRD: green; MOD: orange; MARD: purple). Overlapping protein associations in multiple T2D subtypes are overlaid with the color of each T2D subtype.
